# Nomograms for predicting long-term overall survival and cancer-specific survival in patients with major salivary gland cancer: a population-based study

**DOI:** 10.18632/oncotarget.14905

**Published:** 2017-01-30

**Authors:** Yun Li, Jun Ju, Xiaoxiao Liu, Tao Gao, Zhidong Wang, Qianwei Ni, Chao Ma, Zhenyan Zhao, Yixiong Ren, Moyi Sun

**Affiliations:** ^1^ State Key Laboratory of Military Stomatology, National Clinical Research Center for Oral Diseases, Shaanxi Clinical Research Center for Oral Diseases, Department of Oral and Maxillofacial Surgery, School of Stomatology, Fourth Military Medical University, Xi’an, China; ^2^ Department of Otolaryngology Head and Neck Surgery, Navy General Hospital, Beijing, China; ^3^ Department of Stomatology, Fengtai Hospital, Peking University First Hospital, Beijing, China; ^4^ Department of Stomatology, The First Hospital of Yu Lin, Shaanxi, China; ^5^ Department of Health Statistics, School of Preventive Medicine, Fourth Military Medical University, Xi’an, China

**Keywords:** nomograms, major salivary gland cancer, overall survival, cancer-specific survival, head and neck

## Abstract

In this study, we aimed to develop and validate nomograms for predicting long-term overall survival (OS) and cancer-specific survival (CSS) in major salivary gland cancer (MSGC) patients. These nomograms were developed using a retrospective cohort (*N*=4218) from the Surveillance, Epidemiology, and End Results (SEER) database, and externally validated using an independent data cohort (*N*=244). We used univariate, and multivariate analyses, and cumulative incidence function to select the independent prognostic factors of OS and CSS. Index of concordance (c-index) and calibration plots were used to estimate the nomograms’ predictive accuracy. The median follow-up period was 34 months (1–119 months). Of 4218 MSGC patients, 1320 (31.3%) died by the end of the follow-up; of these 1320 patients, 883 (20.9%) died of MSGC. The OS nomogram, which had a c-index of 0.817, was based on nine variables: age, sex, tumor site, tumor grade, surgery performed, radiation therapy and TNM classifications. The CSS nomogram, which had a c-index of 0.829, was based on the same nine variables plus race. External validation c-indexes were 0.829 and 0.807 for OS and CSS, respectively. Based on SEER database, we have developed nomograms predicting five- and eight-years OS and CSS for MSGC patients with perfect accuracy. These nomograms will help clinicians customize treatment and monitoring strategies in MSGC patients.

## INTRODUCTION

Major salivary gland cancer (MSGC) accounts for approximately 3–6% of all head and neck malignancies [1, 2]. Globally, the overall annual MSGC incidence is 1.195/100,000 [2]. MSGC encompass a cohort of histologies, of which the most common cancer is mucoepidermoid carcinoma, followed by adenoid cystic carcinomas and acinar cell neoplasms, and the most common site of involvement is the parotid gland. Because of the histological heterogeneity and biological behavior diversity, surgery-related issues and the role of radiation therapy were disordered [3, 4], and the 5-year overall survival (OS) rates of MSGC patient vary widely, ranging from 32% to 74% [5, 6]. In addition, since MSGC is rare, most reports of clinical prognostic factors, OS, and cancer-specific survival (CSS) are from small single-institution retrospective studies [5–8]. To our knowledge, no such studies have been performed using data from a national database. The Surveillance, Epidemiology, and End Results (SEER) program of the National Cancer Institute, which collects and publishes cancer incidence and survival data from population-based cancer registries, provides such a free database [9].

Currently, treatment strategies and prognostic predictions for patients with MSGC are based on the American Joint Committee on Cancer (AJCC) TNM staging system (7th edition), which is recommend by the National Comprehensive Cancer Network (NCCN) guidelines [10]. However, the disease staging (I to IV) based on TNM-status dose not take into account the role of other factors that significantly affect the survival of patients with MSGC, such as patient characteristics (age, race, and sex), tumor variables (tumor site, perineural invasion, lymphovascular invasion, and tumor grade) as well as treatment modality (surgery performed and radiation therapy) [5, 6, 8, 11, 12]. Therefore, this system might be inadequate for customized therapeutic decision-making and prognosis prediction, and a new tool is required to address this issue.

Nomograms, which are reliable statistical predictive tools, can estimate individual patient survival with higher accuracy than the AJCC TNM staging system can, by incorporating numerous factors, including TNM elements[13]. Nomograms have been widely developed and applied in a variety of cancers [14–16], and nomograms with good performance have been introduced into NCCN guidelines [17]. Morever, several nomograms have been used to assist clinicians in making treatment and follow-up decisions in patients with head and neck cancers, including squamous cell carcinoma [18–21], adenoid cystic carcinoma [22] and nasopharyngeal cancer [23]. Two studies have reported the utility of nomograms for predicting the outcomes of patients with MSGC [5, 7], but both were based on a single population retrospective cohort and did not include patients who did not undergo surgery.

In the present study, on the basis of multi–institution and multi–population data from SEER database, we aimed to develop the first practical MSGC nomograms that predict long-term OS and CSS. These nomograms can help clinicians design customized treatment and management strategies for patients with MSGC.

## RESULTS

### Patient and tumor characteristics

After applying the screening criteria, 4218 and 244 eligible patients were included in the SEER and Fourth Military Medical University (FMMU) cohort, respectively. The median follow-up period was 34 months (1–119 months) for SEER patients and 50 months (3–120 months) for FMMU patients. Clinical and tumor characteristics are listed in Table 1. The median patient age was 61 years (15–104 years) in the SEER cohort and 59 years (18–96 years) in FMMU cohort. In both cohorts, more than 80% of MSGC originated in the parotid gland, and tended to occur most frequently among older men. The majority of patients in both cohorts had T1–T2 stage (SEER: 56.2%, FMMU: 57.0%), with no node metastasis (SEER: 67.3%, FMMU: 68.9%) and no distant metastasis (SEER: 95.5%, FMMU: 95.1%). In both cohorts, most patients received radiotherapy (SEER: 62.3%, FMMU: 66.4%). By the end of the follow-up period, 1320 (31.3%) patients in the SEER cohort had died, including 883 (20.9%) patients who died from MSGC, and 437 (10.4%) who died of other causes.

**Table 1:** Clinical and tumor characteristics of patients

Variables	SEER Cohort (n=4218)		FMMU Cohort (n=244)	
	No.	%	No.	%
**Age, years**				
15-44	786	18.6	43	17.6
45-54	634	15.1	40	16.4
55-64	874	20.7	54	22.1
65-74	857	20.3	44	18.1
75-84	736	17.5	45	18.4
85+	331	7.8	18	7.4
**Race**				
White	3491	82.8	0	0
Black	372	8.8	0	0
Other*	355	8.4	244	100
**Sex**				
Female	1675	39.7	100	41.0
Male	2543	60.3	144	59.0
**Marital status**				
Unmarried	1122	26.6	50	20.5
Married	3096	73.4	194	79.5
**Grade**				
I	755	17.9	53	21.7
II	1451	34.4	78	32.0
III	1399	33.2	80	32.8
IV	613	14.5	33	13.5
**Laterality**				
Left	2127	50.4	129	52.9
Right	2094	49.6	115	47.1
**Site**				
Parotid	3617	85.8	196	80.3
Submandibular	550	13.0	42	17.2
Sublingual	51	1.2	6	2.5
**T stage**				
T1	1293	30.6	83	34.0
T2	1079	25.6	56	23.0
T3	944	22.4	54	22.1
T4a	737	16.5	37	15.2
T4b	165	3.9	14	5.7
**N stage**				
N0	2838	67.3	148	68.9
N1	590	14.0	32	13.1
N2a	40	0.9	3	1.2
N2b	687	16.3	34	13.9
N2c	27	0.6	3	1.2
N3	36	0.9	4	1.7
**M stage**				
M0	4030	95.5	232	95.1
M1	188	4.5	12	4.9
**Surgery Performed**				
Yes	3971	94.1	244	100
None	247	5.9	0	0
**Radiation**				
Yes	2626	62.3	162	66.4
None	1592	37.7	82	33.6

*Other including American Indian/AK Native, Asian/Pacific Islander.

### Nomograms construction

**Figure 1:**
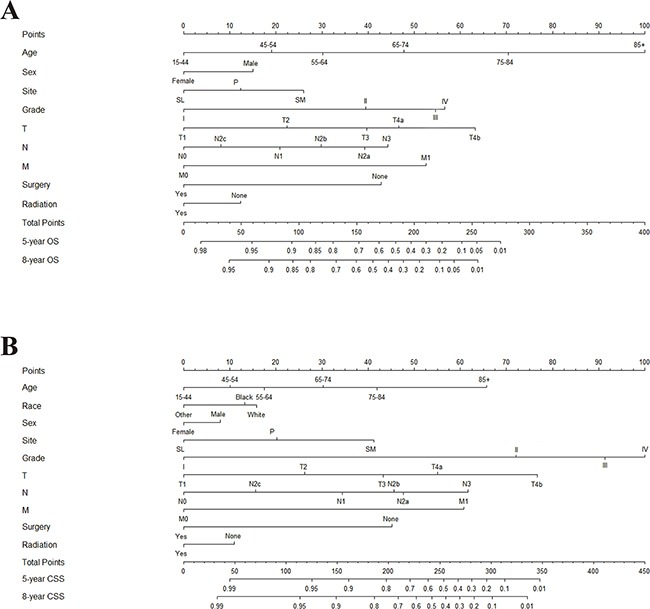
Nomogram for predicting five- and eight-year **A**. overall survival (OS) and **B**. cancer-specific survival (CSS) in patients with MSGC. Abbreviations: Grade: I, Well differentiated; II, Moderately differentiated; III, Poorly differentiated; IV, Undifferentiated. Site: P, parotid gland; SM, submandibular gland; SL, sublingual gland. Race: Other, American Indian/AK Native, Asian/Pacific Islander. Instructions: All the points identified on the points scale for each factor were summed up for each patient. This total point score is identified on the bottom scale to determine the probability of five- and eight-year OS or CSS for an individual patient.

After univariate analysis, all variables other than tumor laterality were found to be statistically associated with OS. Multivariate analyses revealed that nine variables were independent prognostic factors for OS in patients with MSGC: age, sex, tumor site, tumor grade, surgery performed, radiation therapy, and TNM classifications (Table 2). These variables were used to develop the nomogram for predicting five- and eight-year OS (Figure 1A).

**Table 2:** Univariate and multivariate analyses of overall survival in the SEER cohort

Variables	univariate analysis	multivariate analysis	
	*P* Value	HR (95% CI)	*P* Value
**Age, years**	**<0.001**		
15-44		0.126(0.096-0.165)	<0.001
45-54		0.187(0.149-0.235)	<0.001
55-64		0.236(0.195-0.285)	<0.001
65-74		0.339(0.284-0.405)	<0.001
75-84		0.543(0.458-0.643)	<0.001
85+		Reference	
**Race**	**<0.001**		**0.173**
White			
Black			
Other*			
**Sex**	**<0.001**		
Female		0.731(0.646-0.828)	<0.001
Male		Reference	
**Marital status**	**<0.001**		**0.205**
Unmarried			
Married			
**Grade**	**<0.001**		
I		0.308(0.229-0.415)	<0.001
II		0.700(0.589-0.832)	<0.001
III		0.958(0.829-1.107)	0.557
IV		Reference	
**Laterality**	**0.697**		
Left			
Right			
**Site**	**<0.001**		
Parotid		0.752(0.643-0.879)	<0.001
Submandibular		Reference	
Sublingual		0.584(0.286-1.193)	0.140
**T stage**	**<0.001**		
T1		0.269(0.206-0.352)	<0.001
T2		0.430(0.337-0.548)	<0.001
T3		0.615(0.487-0.776)	<0.001
T4a		0.711(0.565-0.894)	0.004
T4b		Reference	
**N stage**	**<0.001**		
N0		0.409(0.265-0.608)	<0.001
N1		0.629(0.406-0.946)	0.026
N2a		0.927(0.509-1.623)	0.747
N2b		0.770(0.490-1.134)	0.170
N2c		0.477(0.256-0.890)	0.020
N3		Reference	
**M stage**	**<0.001**		
M0		0.341(0.281-0.414)	<0.001
M1		Reference	
**Surgery Performed**	**<0.001**		
Yes		0.415(0.350-0.493)	<0.001
None		Reference	
**Radiation**	**<0.001**		
Yes		0.778(0.687-0.880)	<0.001
None		Reference	

*Other including American Indian/AK Native, Asian/Pacific Islander.

Abbreviations: CI; confidence interval; HR, hazard ratio.

The five- and eight-year cumulative incidences of death in the training cohort from MSGC and other causes by are presented clinicopathological variables in Table 3. Gray's test and a multivariate competing risks model revealed ten variables were independent prognostic factors for CSS in patients with MSGC: age, race, sex, tumor site, tumor grade, surgery performed, radiation therapy and TNM classifications. Therefore, a second nomogram predicting five- and eight-year CSS was created using these variables (Figure 1B).

**Table 3:** Five- and eight-year cumulative incidences of death among patients with major salivary gland cancer in the SEER cohort

Variables	Cause-specific death			Death From Other Causes		
	5-year	8-year	*P*	5-year	8-year	*P*
**All Patients**	0.197	0.208		0.084	0.101	
**Age, years**			**<0.001**			**<0.001**
15-44	0.066	0.080		0.010	0.011	
45-54	0.149	0.153		0.024	0.032	
55-64	0.204	0.215		0.044	0.058	
65-74	0.224	0.239		0.077	0.100	
75-84	0.267	0.279		0.168	0.208	
85+	0.353	0.359		0.314	0.329	
**Race**			**<0.001**			**0.001**
White	0.209	0.220		0.090	0.109	
Black	0.142	0.161		0.061	0.067	
Other*	0.135	0.138		0.048	0.062	
**Sex**			**<0.001**			**<0.001**
Female	0.137	0.148		0.052	0.063	
Male	0.237	0.247		0.106	0.127	
**Marital status**			**0.017**			**<0.001**
Unmarried	0.172	0.183		0.053	0.067	
Married	0.206	0.217		0.095	0.114	
**Grade**			**<0.001**			**<0.001**
I	0.021	0.023		0.043	0.052	
II	0.119	0.129		0.064	0.079	
III	0.329	0.344		0.126	0.149	
IV	0.297	0.313		0.088	0.108	
**Laterality**			**0.984**			**0.316**
Left	0.198	0.209		0.089	0.107	
Right	0.196	0.207		0.079	0.095	
**Site**			**<0.001**			**0.371**
Parotid	0.190	0.199		0.086	0.103	
Submandibular	0.254	0.276		0.076	0.092	
Sublingual	0.078	0.098		0.039	0.059	
**T stage**			**<0.001**			**<0.001**
T1	0.064	0.066		0.046	0.058	
T2	0.141	0.151		0.075	0.094	
T3	0.265	0.282		0.118	0.138	
T4a	0.354	0.374		0.127	0.148	
T4b	0.521	0.533		0.061	0.073	
**N stage**			**<0.001**			**0.031**
N0	0.102	0.110		0.075	0.092	
N1	0.333	0.361		0.110	0.127	
N2a	0.425	0.425		0.125	0.150	
N2b	0.429	0.443		0.092	0.109	
N2c	0.481	0.481		0.222	0.222	
N3	0.500	NA		0.083	NA	
**M stage**			**<0.001**			**0.119**
M0	0.174	0.186		0.085	0.103	
M1	0.686	0.686		0.064	0.064	
**Surgery Performed**			**<0.001**			**<0.001**
Yes	0.174	0.185		0.080	0.097	
None	0.571	0.583		0.146	0.164	
**Radiation**			**<0.001**			**0.906**
Yes	0.225	0.239		0.083	0.093	
None	0.152	0.157		0.087	0.099	

*Other including American Indian/AK Native, Asian/Pacific Islander.

### Nomograms validation

**Figure 2:**
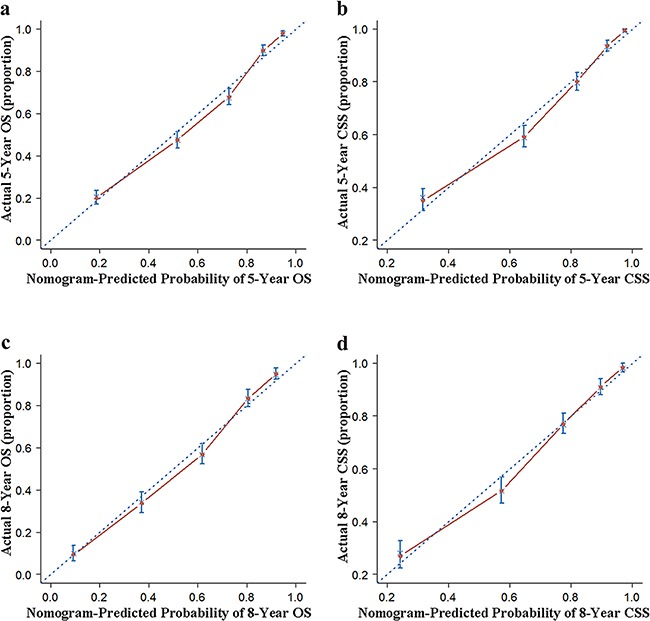
Internal calibration of the nomogram **a**. 5- and **c**. 8-year overall survival (OS) nomogram calibration curves; **b**. 5- and **d**. 8-year cancer-specific survival (CSS) nomogram calibration curves. The dotted line represents the ideal match between the nomogram-predicted (X-axis) and actual survival (Y-axis). Vertical bars indicate 95% confidence intervals.

**Figure 3:**
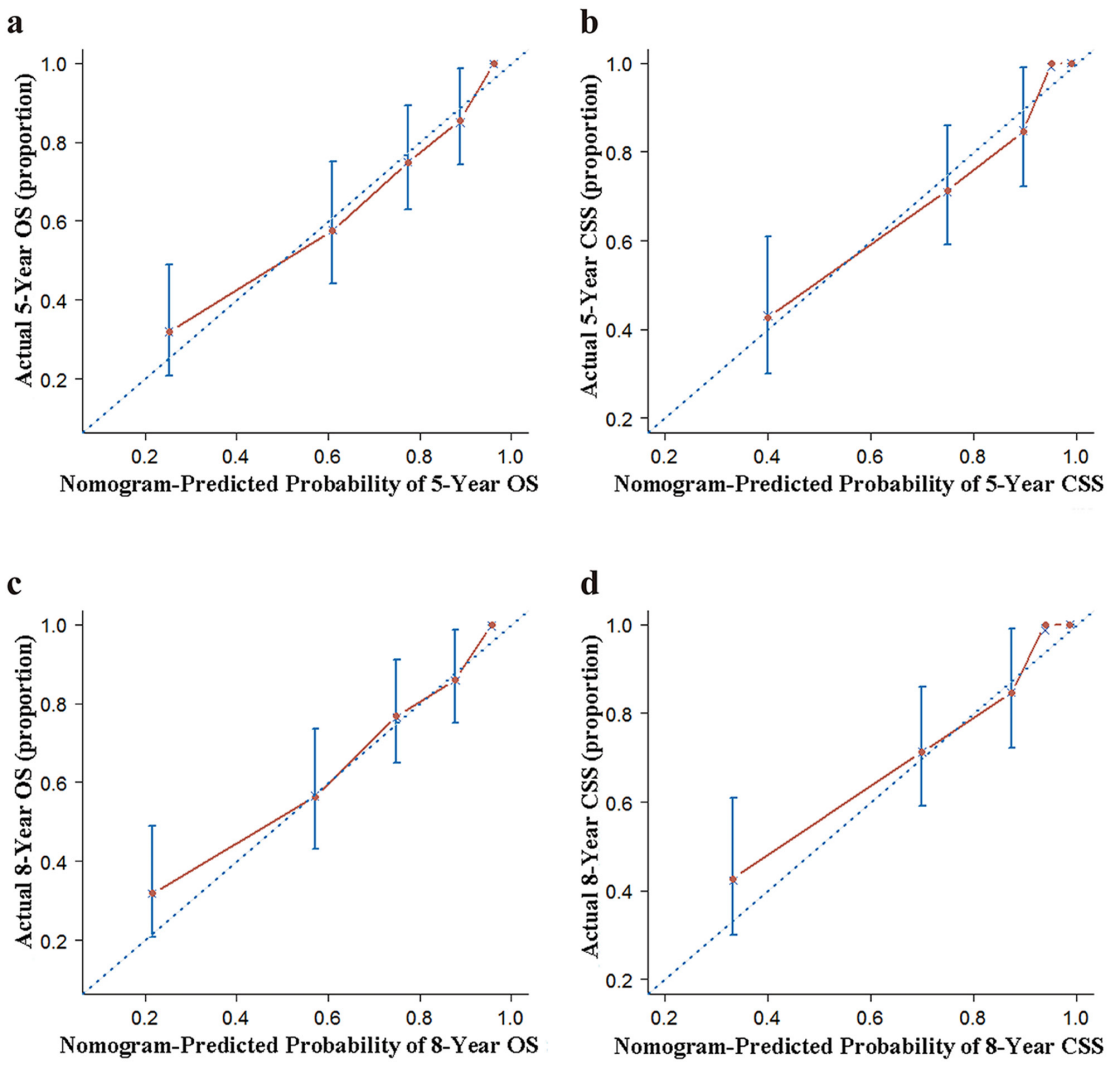
External calibration of the nomogram **a**. 5- and **c**. 8-year overall survival (OS) nomogram calibration curves; **b**. 5- and **d**. 8-year cancer-specific survival (CSS) nomogram calibration curves. The dotted line represents the ideal match between the nomogram-predicted (X-axis) and actual survival (Y-axis). Vertical bars indicate 95% confidence intervals.

In the present study, we performed both internal and external validation of the nomograms. As shown in Table 4, in the internal validation cohort (SEER cohort), models showed good accuracy with c-index of 0.817 (95 % confidence interval [CI], 0.806–0.828) and 0.829 (95 % CI, 0.817–0.841) for MSGC OS and CSS, respectively. External validation using the FMMU cohort showed that the c-index for the OS and CSS nomograms were 0.829 (95 % CI, 0.783–0.869) and 0.807 (95 % CI, 0.761–0.853), respectively. The internal and external calibration curves approached the 45-degree ideal match straight line, indicating that the nomograms for OS and CSS in MSGC were generally well calibrated (Figure 2 and Figure 3).

**Table 4:** The harrell's c-index for the nomogram to predict OS and CSS

Groups	OS		CSS	
	HR	95%CI	HR	95%CI
SEER cohort	0.817	0.806-0.828	0.829	0.817-0.841
FMMU cohort	0.829	0.783-0.869	0.807	0.761-0.853

Abbreviations: OS, overall survival; CSS, cancer-specific survival; HR, hazard ratio; CI, confidence interval.

## DISCUSSION

MSGC is a rare but histologically diverse entity that represents 23 separate primary salivary gland malignant tumors [24]. Although exposure to Ionizing radiation has been reported as a potential causative factor in MSGC development, the specific underlying etiological factors remain unclear [25]. Therefore, the establishment of treatment-related decisions and follow-up strategies for patients with MSGC is challenging but urgently needed. Nomogram is a statistical tool that can meet these requirements. To date, there is no well-designed nomogram for MSGC based on international database. Using the population-based SEER database with a mean follow-up of 34 months, we developed nomograms for predicting five- and eight-year OS and CSS in individual patients with MSGC by applying competing risks analysis.

To ensure the predictive accuracy of nomograms, we used the Kaplan–Meier method and Cox's proportional hazards regression model to select factors for the development of the OS nomogram. A competing risks model was used to select factors for the development of the CSS nomogram. In addition, c-indexes and calibration plots were applied to estimate the predictive accuracy of the models by performing internal and external validation. All nomograms had excellent c-indexes higher than 0.8, and the performance of the calibration plots was ideal.

Compared to the widely accepted TNM staging, our nomograms are not only easy to use, but also have the ability to provide a quantified prognosis for an individual patient. For example, consider two patients with T3N0M0 cancer: case A) a 35-year-old woman diagnosed with moderately-differentiated parotid gland cancer, who underwent both surgery and radiotherapy, and case B) a 60-year-old man diagnosed with undifferentiated submandibular gland cancer, who underwent radiotherapy only. Firstly, a vertical line is drawn from every factor to the “Points” line in the nomogram. Second, all the “Points” are summed up to obtain the “Total Points” and a vertical line is drawn from “Total Points” to the “OS” and “CSS” line to obtain corresponding survival. Thus, used nomograms descried in the present study reveal that the patients in case A and B have eight year OS probabilities of 86% and 30%, respectively, and eight year CSS probabilities of 94% and 45%, respectively. However, according to TNM staging [26], both patients would be classified as stage III, which indicating identical outcomes.

In the present study, several clinical and pathologic characteristics were shown to be independent prognostic factors for OS and CSS in patients with MSGC, including age, sex, tumor site, tumor grade, surgery performed, radiation therapy, and TNM classifications, which is consistent with previous reports [5, 6, 8, 11, 27–30]. Apart from these, comorbidity and postoperative complications, which were not included in the present study, have been proven to be accurate prognostic factors [11, 30]. However, data from previous studies have revealed that a higher incidence of comorbidity and complications is significantly associated with advanced age [30, 31]. Thus, this limitation was potentially compensated by the effect of advanced age on mortality in the models.

Interestingly, on the CSS, we found that both 5- and 8-year cause-specific death (CSD) rates of MSGC patients who did not received radiotherapy (15.2% and 15.7%, respectively) were lower than those of MSGC patients who received radiotherapy (22.5% and 23.9%, respectively). In contrast, on the OS, radiotherapy performed improved the 5- and 8-year OS of patient with MSGC. This may be explained as follows: First, whereas 1320 of the 4218 patients with MSGC died of MSGC-related causes, 437 (33.1%) died of other causes other than MSGC, and 5- and 8-year mortality rates from other causes other than MSGC decreased with radiotherapy from 8.7% to 8.3% and 9.9% to 9.3%, respectively. In addition, radiotherapy was primarily used in patients with higher-grade disease or as a treatment option for advanced inoperable salivary gland tumors [6, 21].

The present study has several merits. Our nomograms have excellent accuracy with an overall c-index>0.80, which compares very favorably with those of other widely accepted nomograms in other cancers [15, 18–21], whose c-indexes range from 0.60 to 0.80. Moreover, the variables utilized in these nomograms are easily available in clinical practice. Finally, compared with previous MSGC nomograms, the present nomograms were developed on the basis of information from an international database, and were externally validated by using another independent cohort (FMMU cohort).

Despite these merits, the present study also has some limitations. First, our nomograms were constructed using retrospective data, which introduces the risk of potential selection bias. In addition, data on some important clinicopathological variables were incomplete, reducing the number of eligible case. Second, although the quality of SEER database information is considered high, TNM classifications information was not available until 2004, and the prognostic factor lymph-vascular invasion [32] was not included until 2010. Therefore, we failed to predict a survival time longer than eight years and lymph-vascular invasion was not included in the nomograms, but we plane to address these limitation in a future study. Third, data regarding tumor recurrence, chemotherapy [33], and perineural invasion [32], which are important prognostic factors for MSGC, is not available in the SEER database. Therefore, we failed to collect and analyze these factors, and develop nomograms for predicting loco-regional control.

In summary, based on a large population-based cohort, we have developed and externally validated two clinically useful nomograms that could objectively provide five- and eight-year OS and CSS for patients with MSGC for the first time. The performance of these nomograms was accurate and they may aid patient counselling, clinical decision-making, and the development of follow-up strategies for management of MSGC.

## MATERIALS AND METHODS

### SEER cohort

**Figure 4:**
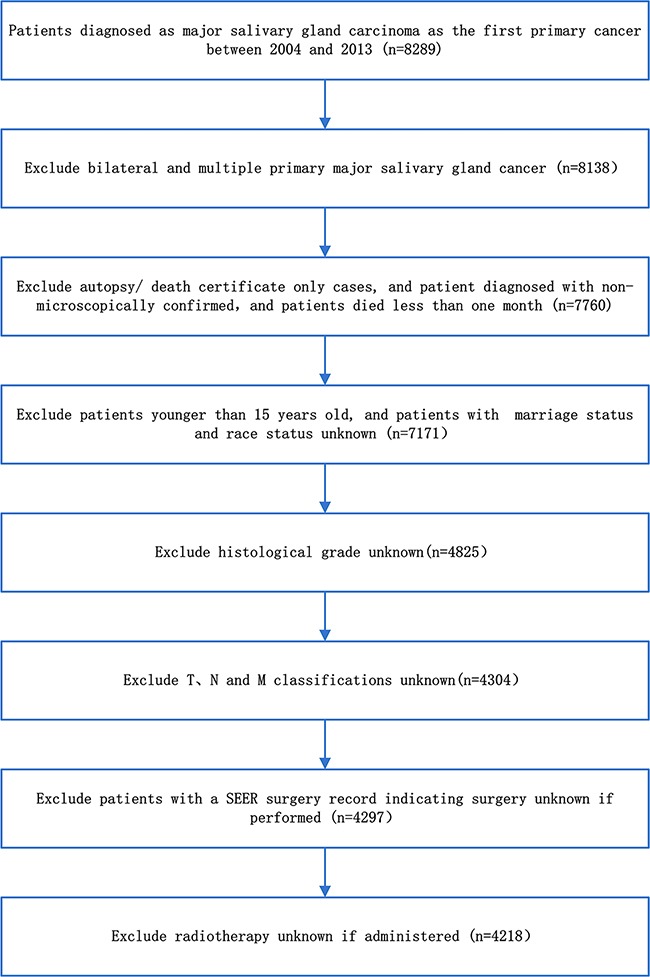
The flow diagram of data selection for the SEER cohort

We identified information on the clinical and pathologic characteristics of all patients diagnosed with major salivary gland carcinoma between 2004 and 2013 from the SEER program of the National Cancer Institute, which is a national collaboration program [9]. The flow diagram of data selection is shown in Figure 4. Briefly, the basic inclusion criteria were as follows the primary tumor site was major salivary gland, including the parotid, submandibular, and sublingual glands; malignant behavior; and age older than 15 years at diagnosis. To improve the accuracy and homogeneity of the SEER cohort, the final inclusion criteria were as follows: diagnostic information confirmed microscopically and not from a death certificate or autopsy only; active follow-up; patient and tumor information (age, race, sex, marital status, tumor site, tumor laterality, tumor grade, surgery performed, radiation therapy and TNM classifications) were known and exact. A total of 4017 patients were excluded due to indefinite follow-up information or because patients died less than 1 month after treatment. After applying the screen criteria, 4218 patients were included in the final SEER cohort.

For analyses, age was transformed into categorical variables on the basis of recognized cutoff values. Race classification based on the SEER Program was as follows: white, black, and other (American Indian/Alaskan Native and Asian/Pacific Islanders). Tumor grade included I (well differentiated), II (moderately differentiated), III (poorly differentiated) and IV (undifferentiated). All patients TNM classification were staged according to the 7th edition AJCC Staging Manual [26].

### FMMU cohort

The FMMU cohort comprised 256 patients who were histologically diagnosed with major salivary gland carcinoma at the department of Oral and Maxillofacial Surgery, School of Stomatology, Fourth Military Medical University (FMMU) in China. All inclusion criteria were identical to those used in the SEER cohort except that all patients were Chinese and received surgery as the primary treatment. Twelve patients were excluded, eight patients because of indefinite follow-up information, and four because they died less than 1 month after surgery. After applying the screen criteria, 244 patients were included in the final FMMU cohort.

### Nomograms

The SEER cohort was used to establish the OS and CSS nomogram. OS was defined as the time from diagnosis to death or censoring (if a patient was alive at the last follow-up). The median follow-up time was estimated as the actual patient survival time. The Kaplan-Meier method and log-rank test were used to conduct the univariate prognostic analysis. Variables that were possible prognostic factors (*P* < 0.001) on univariate analyses were included in the multivariate cox proportional hazards analysis to yield independent MSGC OS factors (*P* < 0.001) [34]. Next, the nine independent prognostic factors in multivariate analyses were used to build nomogram for five- and eight- year OS in patients with MSGC at by employing a stepwise-selection method in the R software.

When constructing a competing risks nomogram for MSGC, death from MSGC and death from other causes were considered two different event types in this analysis. CSS was defined as the time from diagnosis to death attributed to MSGC or censoring (if a patient was alive at the last follow-up or death from other causes). The cumulative incidence function (CIF) was used to assess the probability of death, and the difference was assessed using Gray's test [35]. Variables whose *P* values were less than 0.001 for the CIF values were considered significant independent MSGC CSS factors. Subsequently, by integrating all the significant independent factors, we developed nomograms to predict five- and eight-year CSS in patients with MSGC via a proportional sub-distribution hazards regression method proposed by Fine and Gray [36] using R software“cph” and “step” commands.

### Nomograms validation

The SEER and FMMU cohorts were applied to estimate the predictive accuracy of the model by performing internal and external validation, respectively. All the internal and external validations were measured by c -index and calibration plots, and performed using bootstrapping with 1000 resamples and ten-fold cross-validation, respectively. C-indexes quantified the discrimination between predicted and actual situations, with values ranging from 0.5 (no discrimination) to 1.0 (perfect discrimination), proposed by Harrell [37]. In addition, a marginal estimate versus model was used to plot calibration curve that represented the calibration between nomogram-predicted and actual survival.

All statistics analysis was conducted using the SPSS software version 19.0, (SPSS Inc., Chicago, IL, USA) and the R software version 3.3.0 (R Foundation for Statistical Computing, Vienna, Austria; www.R-project.org) with the R packages rms, and cmprsk. All calculated *P* values were two sided, and *P* <0.001 was considered statistically significant.

### Ethics statement

Our study was approved by the Fourth Military Medical University Ethical Committee. Informed patient consent was not required for data released by the SEER database.
